# Direct alcohol biomarkers in blood samples of traffic offenders - correlation between blood alcohol concentration, ethyl glucuronide, and phosphatidylethanol

**DOI:** 10.1007/s00414-026-03834-w

**Published:** 2026-05-19

**Authors:** Anna Holzer, Jasna Neumann, Andreas Stoever, Matthias Graw, Sylvia Schick, Anouk Ludwig, Michael Boettcher, Benno Hartung

**Affiliations:** 1 Institute of Legal Medicine, University Hospital Essen, Essen, Germany; 2 MVZ Medizinische Labore Dessau Kassel GmbH, Dessau, Germany; 3 Institute of Legal Medicine, Ludwig-Maximilians-University Munich, Munich, Germany

**Keywords:** Phosphatidylethanol (PEth), Ethyl glucuronide (EtG), Blood alcohol concentration (BAC), Direct alcohol biomarkers, Alcohol consumption patterns

## Abstract

**Background:**

Phosphatidylethanol (PEth) and ethyl glucuronide (EtG) are established biomarkers of alcohol consumption, offering complementary insights into long-term and recent drinking behavior, as well as alcohol tolerance or dependence. This study examined the association between blood alcohol concentration (BAC), EtG, and PEth in blood samples collected predominantly in traffic-related forensic cases.

**Methods:**

A total of 100 consecutive blood samples from 87 individuals submitted to the Institute of Legal Medicine in Munich for BAC determination were retrospectively analyzed for PEth and EtG. Samples originated from individuals in North Rhine-Westphalia, including mainly driving under influence (DUI), but also other alcohol-related offenses. BAC was measured via gas chromatography with a flame ionization detector and an enzymatic (ADH) method. PEth and EtG were quantified by validated LC-MS/MS procedures.

**Results:**

Thirteen samples were excluded as post-drinking pairs from the same individuals, resulting in *n* = 87 samples included in the analysis. The study population was predominantly male (77%), with a mean age of 37 years. Most cases involved traffic offenders resp. drivers of motor vehicles, primarily cars. BACs were ≥ 1.1 g/kg in 73.5% and ≥ 1.6 g/kg in 47.1% of cases. EtG concentrations ranged from below cutoff to 8,627 ng/mL (median: 2,957 ng/mL), and PEth ranged from below the lower limit of quantification (LLOQ) to 1,520 ng/mL (median: 140 ng/mL). A moderate correlation was found between BAC and PEth (*r* = 0.48, *p* < 0.01), and a strong correlation between BAC and EtG (*r* = 0.79, *p* < 0.001). Both BAC and EtG concentrations increased with rising PEth categories, suggesting an association between acute alcohol intoxication and patterns of repeated consumption.

**Conclusion:**

PEth findings suggest that alcohol-related driving offenses often involve repeated or heavy alcohol consumption. Combined EtG, PEth, and BAC analysis offers a comprehensive approach to distinguish between single drinking episodes and habitual use, and is particularly useful for evaluating isolated drinking after prolonged abstinence.

## Introduction

The objective assessment of alcohol consumption patterns is essential in traffic medicine, particularly in the context of legal driving fitness evaluations under § 13 of the German Driving License Ordinance (FeV). Conventional approaches, such as self-reported drinking behavior, clinical interviews, and indirect biochemical markers like γ-glutamyltransferase (γ-GT) or carbohydrate-deficient transferrin (CDT), are limited by low sensitivity and high susceptibility to bias [1–3].

In recent years, direct ethanol biomarkers such as ethyl glucuronide (EtG) and phosphaditylethanol (PEth) have gained attention due to their higher specificity and ability to reliably reflect alcohol intake. EtG is a non-oxidative phase II metabolite of ethanol formed via conjugation with glucuronic acid. It can be measured in various matrices, including urine, blood, and hair. In blood, EtG reflects recent alcohol consumption within hours depending on the amount ingested and individual metabolism [3]. Compared to urine, blood EtG concentrations are less influenced by renal function or urine dilution, and may therefore provide a more controlled window of detection in forensic settings, if sensitivity is increased adequately [1].

PEth, in contrast, means a group of phospholipid homologues formed exclusively in the presence of ethanol through the action of phospholipase D. It accumulates in red blood cell membranes and reflects repeated or chronic ethanol exposure, providing a detection window of up to several weeks [4]. Especially PEth 16:0/18:1 is highly specific to ethanol and is considered one of the most sensitive biomarkers for long-term alcohol intake [3, 4].

In Germany, legally and medically relevant cut-off concentrations have been established to support forensic and traffic medicine evaluations. For EtG in urine, a threshold of 100 ng/mL is widely accepted to indicate recent consumption, whereas concentrations below 50 ng/mL are commonly used to confirm abstinence. A cutoff of 1 ng/mL for EtG in blood is considered comparable to the forensic threshold of 100 ng/ml in urine [5]. For EtG in whole blood, concentrations above 5 ng/mL are typically interpreted as indicative of recent alcohol intake [1]. For PEth, a concentration of ≥ 20 ng/mL is used to spot single to repeated alcohol consumption, while concentrations above 200 ng/mL may suggest harmful or chronic intake [6, 7]. In Germany, an upper cutoff of 210 ng/ml is recommended for forensic cases [8].

In forensic and traffic medicine practice, expert witnesses are occasionally confronted with cases where individuals present with markedly elevated blood alcohol concentrations (BAC). In such instances, defendants often claim that the elevated BAC stems from a single, exceptional drinking episode, as hints of alcohol abuse could jeopardize the driver’s license. However, BAC reflects only the acute, real-time ethanol level and does usually not provide retrospective insight into an individual’s habitual drinking behavior.

Schröck et al. [9] demonstrated that PEth and EtG provide complementary information in alcohol sober individuals, with PEth better suited to detect habitual alcohol use and EtG reflecting recent consumption. Their findings support the notion that a combined analysis of both markers could improve the differentiation of individual drinking patterns.

Against this background, the present study investigates the relationship between BAC, PEth, and EtG in blood samples collected primarily in the context of traffic offenses under the acute influence of alcohol. The aim was to evaluate whether alcoholised drivers/individuals are generally likely to be social or heavy drinkers.

## Materials and methods

A total of 100 consecutive blood samples submitted to the Institute of Legal Medicine in Munich for solely BAC analysis in November 2022 were retrospectively examined for PEth and EtG.

The samples were collected in the federal state of North Rhine-Westphalia and included cases of driving under the influence (DUI) as well as other offenses suspected to involve alcohol impairment. Upon delivery to the Munich laboratory, the blood samples were aliquoted with permission from the Ministry of the Interior of North Rhine-Westphalia (LZPD NRW) and the state authorities.

One aliquot of each sample was analyzed for BAC at the Institute in Munich according to the German forensic guidelines. Four independent measurements were conducted using both gas chromatography with a flame ionization detector and enzymatic (alcohol dehydrogenase, ADH) methods, and the arithmetic mean of the four concentrations was, after converting from g/L plasma to g/kg whole blood (‰), reported as the BAC [10].

A second aliquot of each sample was frozen to -80 °C and sent cooled to the MVZ Medizinische Labore Dessau Kassel GmbH, a laboratory in Dessau for quantification of PEth and EtG. Samples were stored at − 80 °C after arrival until analysis. Quantification of PEth and EtG was performed with two different LC-MS/MS methods. Both LC-MS/MS methods were accredited according to ISO 15,189 and ISO 17,025 and were used for clinical and forensic routine samples. Measuring range for PEth was between 10 ng/mL and 5,000 ng/mL with a lower limit of quantification (LLOQ) of 9.1 ng/mL. The measuring range for EtG in whole blood was between 0.6 and 5,000 ng/mL with a cutoff of 1 ng/mL Samples above the measuring range were tested again after dilution.

General sociodemographic data such as sex, age, offense type, and vehicle category were extracted from the accompanying police request forms submitted with the samples.

To prevent potential re-identification of cases, BAC values were grouped. In Germany, several thresholds apply to DUI. A BAC of ≥ 1.1 g/kg constitutes a criminal offense regardless of observable signs of impairment and results in the loss of the driver`s license. For BACs between 0.5 g/kg and 1.1 g/kg, a criminal offense may apply if the driver shows signs of impairment or alcohol-related driving errors; otherwise, the act is considered an administrative offense. When BACs exceed 1.6 g/kg, offenders are required to demonstrate their fitness to drive.

The dataset included 13 cases of suspected drinking after driving, in which two blood samples of the same individual were collected at intervals of 30 to 40 min. In these cases, the first sample was used for this evaluation, with one exception, where BAC determination failed in the first sample due to sample quality. In this case, the second sample was used for evaluation. The remaining 13 samples were excluded, resulting in 87 blood samples included in the analysis.

### Ethics statement

Ethical approval for this study was granted by the Ethics Committee of Ludwig-Maximilians-University of Munich, Germany (approval number 22–0664). The use of forensic blood samples was additionally approved by the Ministry of the Interior of North Rhine–Westphalia. The analysis was performed in accordance with the Declaration of Helsinki and all relevant guidelines and regulations. Individual consent to participate was not required.

### Statistical analysis

All data were anonymized prior to analysis and evaluated using standard spreadsheet software (Xact, Hamburg 2004), Microsoft Office LTSC Professional Plus, excel Version 2408 (Microsoft^®^ Excel^®^ LTSC MSO (Version 2408 Build 16.0.17932.20602) 64 Bit), and IBM SPSS Statistics, Version 29.0.0.

Descriptive and explorative statistics like cross tables for frequencies and calculations of median, minimum, maximum, and mean were performed depending on variable scale. Pearson correlations coefficients between continuous variables are independently calculated and presented in addition. Scatterplots are used to show the common distributions of BAC values with EtG and PEth values, respectively; bar charts are used for sample number description and frequencies of PEth groups among BAC groups, and boxplots are utilized to visualize BAC and EtG value distributions for PEth groups.

## Results

### Overall cohort

Excluding 13 samples due to paired consecutive blood samples in drinking-after-driving cases, the study included 87 individuals, with 77 males and 10 females. The mean age of the subjects was 37 years (median: 33 years, range: 17–74 years).

**Fig. 1:**
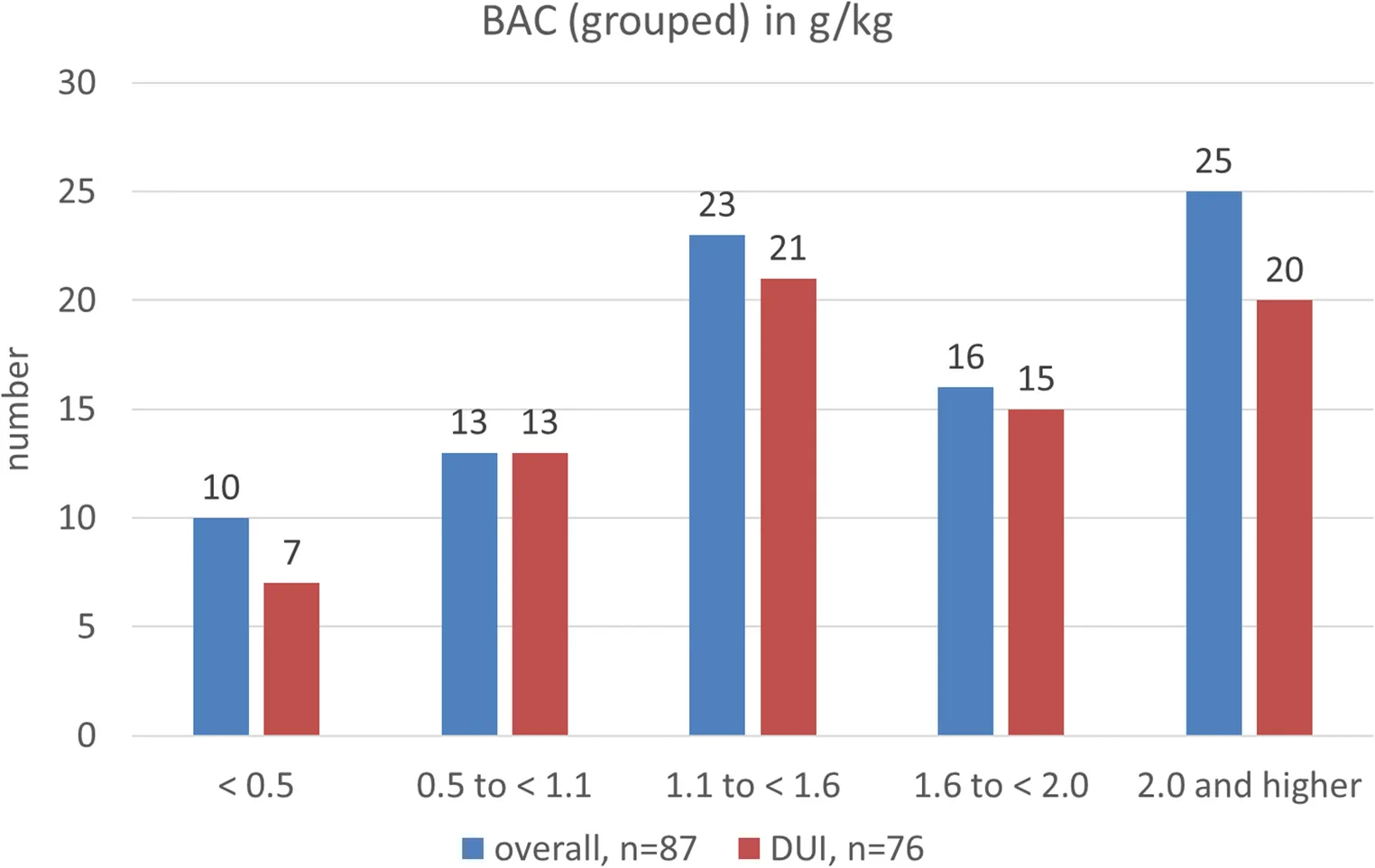
Absolute frequencies of grouped blood alcohol concentrations (BACs) in g/kg for the overall cohort and the driving-under-influence (DUI) cohort

BAC analysis revealed that 64 individuals (73.5%) had a BAC ≥ 1.1 g/kg, corresponding to the legal threshold for absolute driving incapacity in Germany. Among them, 41 exhibited BACs ≥ 1.6 g/kg (47.1%), while 10 individuals had a BAC below 0.5 g/kg and three individuals tested negative for alcohol (Fig. 1).

The median blood concentration of EtG was 2,975 ng/mL (range: above cutoff of 1 ng/mL to 8,627 ng/mL, mean: 2,911 ng/ml), with 96.6% of cases exceeding 100 ng/ml. PEth 16:0/18:1 concentrations showed a median of 140 ng/mL, ranging from LLOQ (9.1 ng/ml) to 1,520 ng/mL (mean: 252 ng/ml). In total, 35 individuals (40.2%) exhibited PEth concentrations ≥ 200 ng/ml and 3 cases were below 20 ng/ml.

Of the three individuals with negative BAC results, two also had EtG and PEth concentrations below the respective detection limits. The third individual, despite a negative BAC, showed a PEth concentration of 99.4 ng/mL and an EtG concentration of 35.6 ng/mL.

**Fig. 2:**
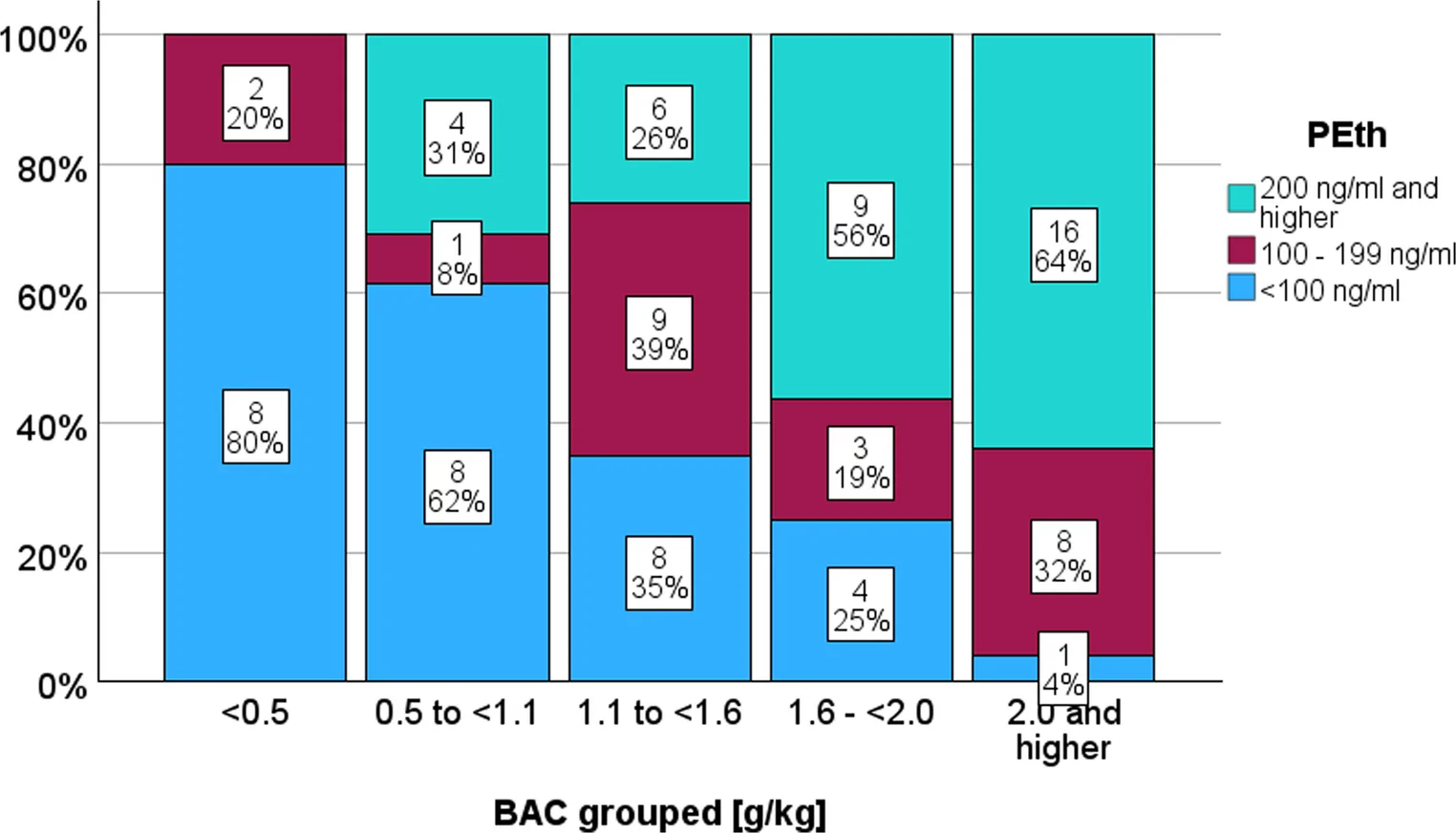
Absolute and relative distribution of PEth groups by BAC groups in the overall cohort (*n* = 87)

None of the individuals with BACs below 0.5 g/kg had PEth concentrations ≥ 200 ng/mL, whereas 31 of 64 (48%) individuals with BACs ≥ 1.1 g/kg did. Among the 41 individuals with BAC ≥ 1.6 g/kg, there were 25 (61%) individuals with PEth concentrations above 200 ng/ml, but still five (12%) with PEth values below 100 ng/ml, see also Fig. 2.

**Fig. 3:**
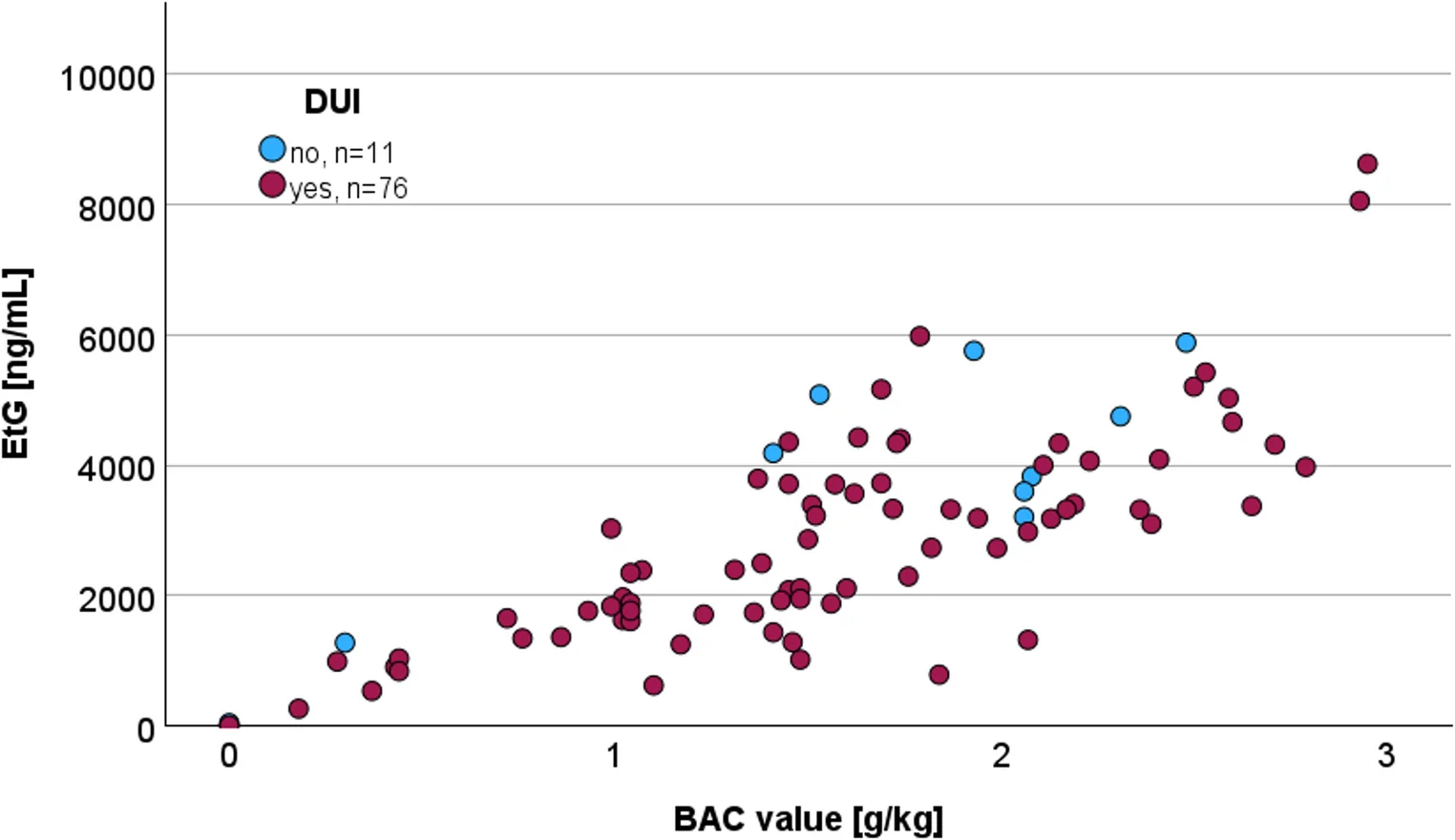
Scatterplot of BAC (g/kg) and EtG (ng/mL), with DUI cases highlighted in red

**Fig. 4:**
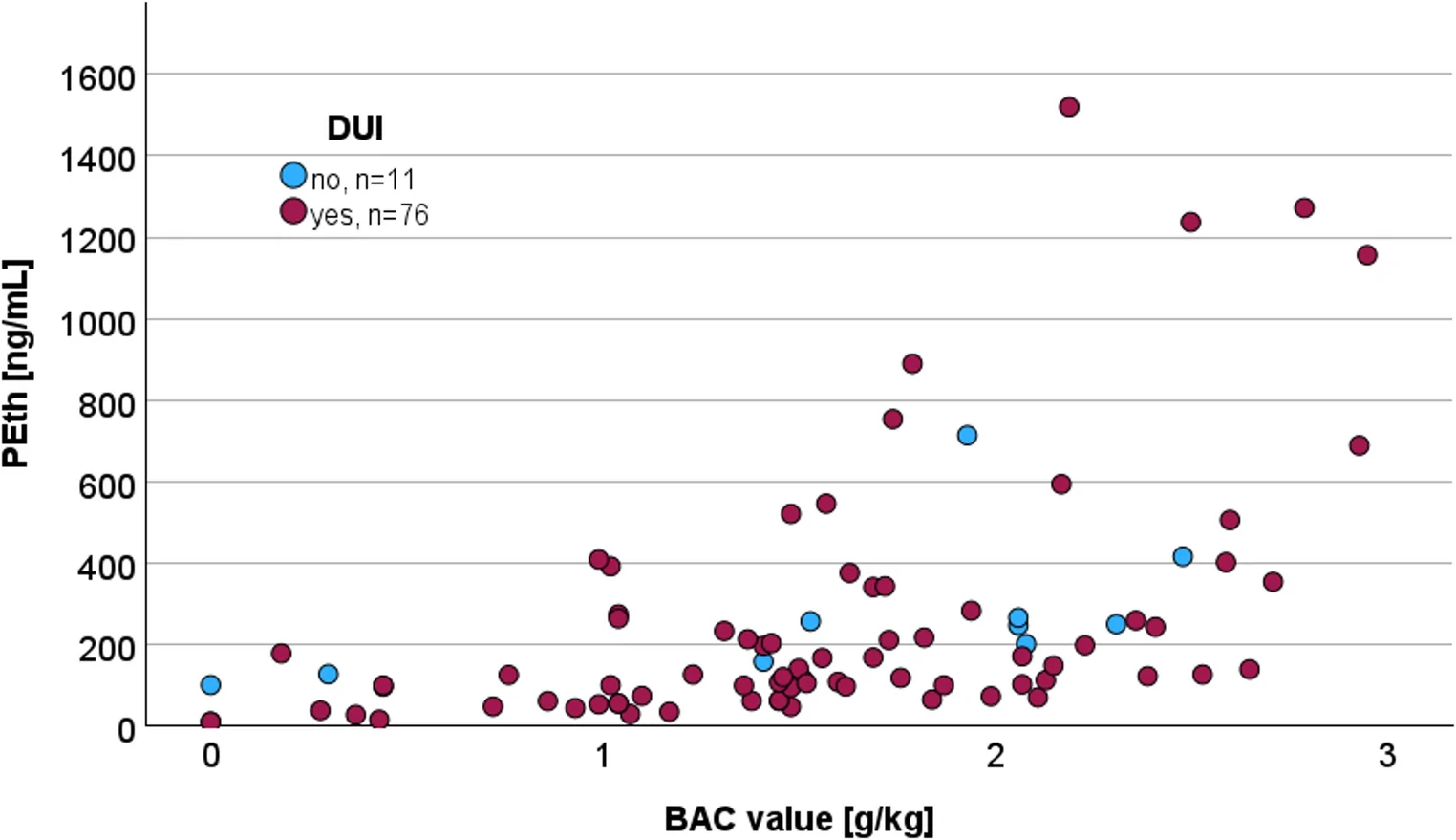
Scatterplot of BAC (g/kg) and PEth (ng/ml), with DUI cases highlighted in red

A strong positive correlation was observed between BAC and EtG concentrations in blood (*r* = 0.778, *p* < 0.01), confirming the close relationship between acute alcohol intake and EtG concentrations (Fig. 3). In contrast, BAC and PEth concentrations showed a moderate correlation (*r* = 0.497, *p* < 0.01) (see Fig. 4), suggesting that although PEth concentrations increase with BAC, individual variability in drinking patterns likely modulates the strength of this relationship.

**Fig. 5:**
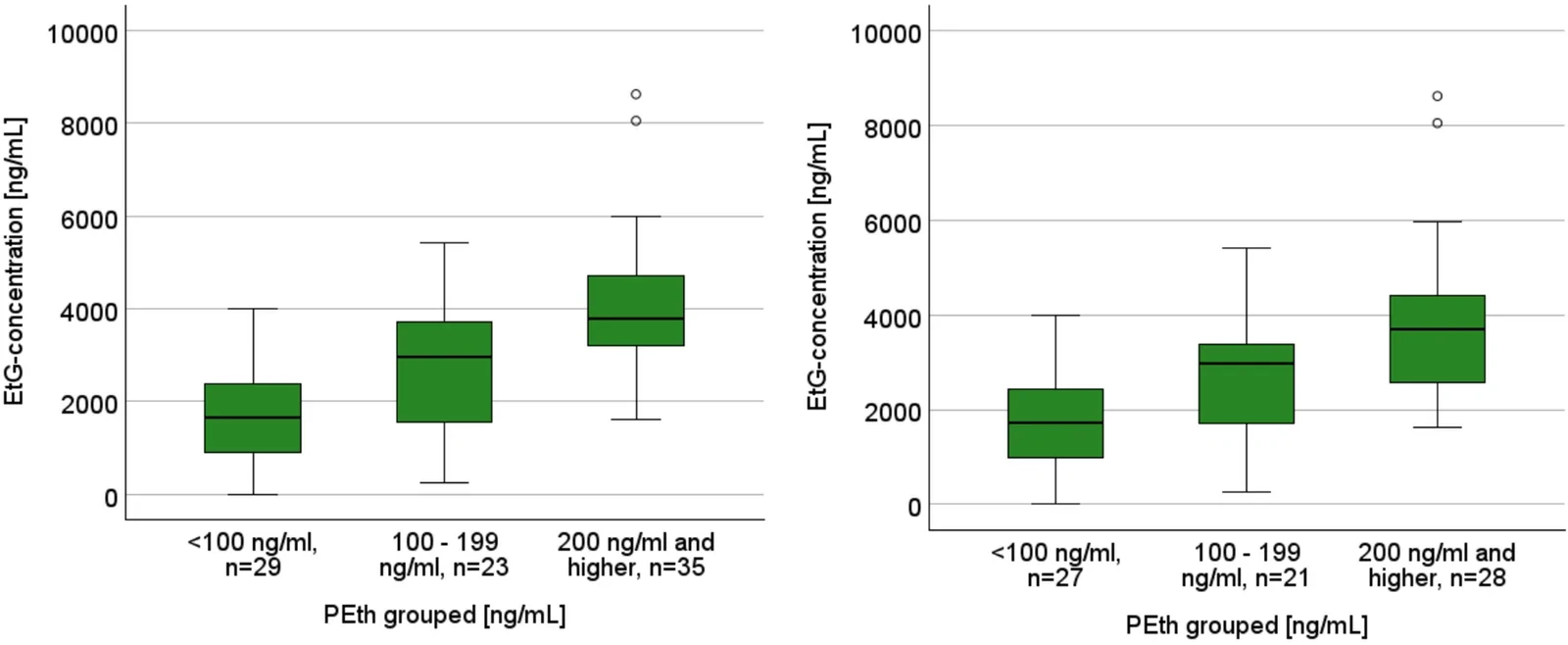
Distribution of EtG concentrations by PEth groups: left panel shows the overall sample (*n* = 87), right panel shows DUI cases (*n* = 76)

**Fig. 6:**
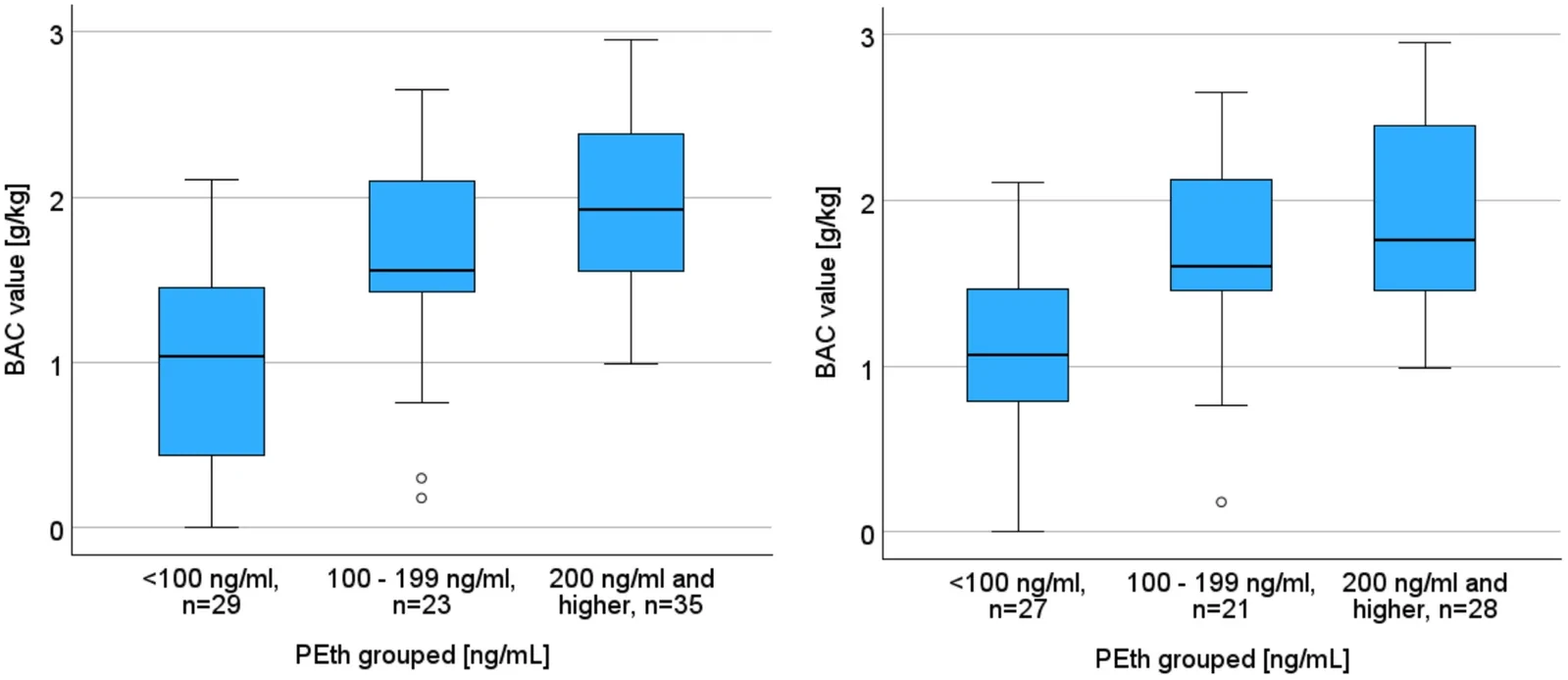
Distributions of BACs by PEth groups: left panel shows teh overall sample (*n* = 87), right panel shows DUI cases (*n* = 76)

To evaluate PEth not only as a marker of chronic alcohol use but also as a potential indicator of recent or acute consumption, EtG and BAC were analyzed across incremental PEth categories (< 100 ng/mL, 100–199 ng/mL, ≥ 200 ng/mL), as shown in Figs. 5 and 6.

In the PEth < 100 ng/mL group, the median EtG concentration was 1,647 ng/mL (mean ± SD: 1,716 ± 1,121 ng/ml), and the median BAC was 1.04 g/kg (mean ± SD: 1.03 ± 0.6). Concentrations were more heterogeneous in this group, with EtG ranging from below the cutoff to 3,998 ng/mL and BAC from 0 to 2.11 g/kg, indicating substantial variability in recent drinking behavior.

In the PEth 100 – <200 ng/mL group, median EtG increased to 2,975 ng/mL (mean ± SD: 2,803 ± 1,381 ng/ml), and the median BAC to 1.56 g/kg (mean ± SD: 1.63 ± 0.63 g/kg). Maximum concentrations reached 5,422 ng/mL for EtG and 2.65 g/kg for BAC, with two BAC outliers (0.18 and 0.3 g/kg) observed.

In the PEth ≥ 200 ng/mL group, both markers reached their highest concentrations, with a median EtG of 3,789 ng/mL (mean ± SD: 3,972 ± 1,623 ng/ml) and a median BAC of 1.93 g/kg (mean ± SD: 1.94 ± 0.56 g/kg). Maximum concentrations were 8,627 ng/mL for EtG and 2.95 g/kg for BAC.

### DUI-cases

Of the 87 individuals, 76 were involved in DUI incidents, while the remaining 11 were suspected of non-traffic-related offenses. Among DUI cases, the majority operated cars (*n* = 54), followed by bicycles/e-bikes (*n* = 11), e-scooters (*n* = 6), mopeds (*n* = 3), and motorcycles (*n* = 2).

Within the DUI group, 73.7% of individuals (*n* = 56) exceeded the legal threshold for absolute driving incapacity in Germany (BAC ≥ 1.1 g/kg), and 46.1% (*n* = 35) exhibited BACs ≥ 1.6 g/kg Conversely, 9.2% (*n* = 7) had BACs below the administrative offence limit of 0.5 g/kg, including three individuals with BACs < 0.3 g/kg (see Fig. 1).

The median EtG concentration among DUI cases was 2,608 ng/mL (range: below cutoff – 8,627 ng/mL). The median PEth concentration was 125 ng/mL (range: below cutoff − 1,520 ng/mL), with 36.8% of individuals (*n* = 28) showing PEth ≥ 200 ng/ml.

Of the 35 individuals within the DUI group with BACs ≥ 1.6 g/kg — and thus required to undergo an assessment of fitness to drive — 54.3% showed PEth concentrations ≥ 200 ng/mL, indicating drinking patterns inconsistent with a single episode of excessive alcohol intake. Only five (14.3%) in this group showed PEth concentrations < 100 ng/ml. The median EtG concentration in this group was 3,720 ng/mL (range: 780–8,627 ng/mL).

Correlations among BAC, EtG and PEth in the DUI subgroup were consistent with those in the overall cohort showing a strong positive correlation between BAC and EtG (*r* = 0.767), and moderate correlations between BAC and PEth (*r* = 0.502) as well as EtG and PEth (*r* = 0.556).

## Discussion

This study investigated the relationship between BAC, EtG, and PEth with a focus on traffic offenders. The results demonstrate a consistent association between acute alcohol intoxication, recent drinking behavior, and long-term alcohol use, as measured by BAC, EtG, and PEth, respectively.

A strong positive correlation between BAC and EtG (*r* = 0.778, *p* < 0.01) confirms EtG as a reliable biomarker for recent alcohol intake, typically within the past few hours [3]. The gradual increase in EtG concentrations across increasing PEth categories holds limited significance during acute intoxication, as the duration of intoxication among the subjects is unknown and EtG levels continue to rise with ongoing alcohol metabolism. Nevertheless, these patterns support the use of EtG and PEth as complementary biomarkers for assessing different temporal dimensions of alcohol intake — a concept further substantiated by Neumann et al., who demonstrated that the combined measurement of PEth, EtG, and ethanol in blood provides a more robust characterization of drinking behavior in both clinical and forensic settings [11].

PEth concentrations showed a moderate correlation with BAC (*r* = 0.497, *p* < 0.01), and median BAC increased from 1.04 g/kg in individuals with low PEth levels to 1.93 g/kg in those with PEth ≥ 200 ng/mL. This may reflect that individuals with evidence of habitual drinking also tend to present with higher concentrations of acute intoxication. However, overlapping BACs within PEth categories underscore the influence of individual factors such as tolerance, drinking pattern, and timing of last consumption. Supporting these findings, Schröck et al. demonstrated that PEth analysis in blood can help distinguish between social drinking and excessive alcohol use in DUI cases [9]. Similarly, Dağlıoğlu et al. reported a strong positive correlation between PEth and BAC (*r* = 0.784) in traffic accident cases, highlighting the utility of PEth — particularly in combination with EtG — for the forensic evaluation of alcohol consumption [12].

Studies showed that even low to moderate drinking events in previously abstinent individuals can yield PEth concentrations above 20 ng/mL [13, 14]. Similarly, Spleis et al. demonstrated that consumption of 0.6–0.75 g/kg ethanol can result in PEth concentrations above common forensic thresholds, depending on drinking context and metabolism [15]. These findings underline the importance of interpreting PEth concentrations carefully, especially when considering abstinence verification or differentiating one-time from repeated use.

In Germany, suspects of DUI are required to provide a blood sample if breath alcohol testing is refused, not feasible or if individuals are suspected of having committed an alcohol related criminal offense. In such cases the combined analysis of BAC, EtG, and PEth provides greater diagnostic depth than any single marker alone. While BAC reflects only acute intoxication and does not account for drinking patterns, this becomes particularly relevant when high BACs are observed without corresponding clinical signs of intoxication, suggesting alcohol tolerance or chronic consumption. The inclusion of PEth and EtG allows experts to evaluate whether a high BAC is the result of on-time intake or habitual drinking— an essential distinction in both criminal liability and driver`s licensing assessments.

In Germany, PEth concentrations ≥ 20 ng/mL are considered indicative of recent drinking, while concentrations ≥ 200 ng/mL in clinical context [6] resp. ≥210 ng/mL in forensic cases [8] suggest chronic use. EtG concentrations > 5 ng/mL in blood are widely accepted as indication of recent alcohol consumption. Most individuals in this study exceeded one or both thresholds, indicating substantial alcohol use among traffic offenders and supporting the integration of PEth and EtG testing into routine forensic assessments.

In 2023, about 657,000 verdicts were issued by German criminal courts, with roughly 17% related to traffic offenses (e.g., § 316 German criminal code) [16]. Bavarian data show that around 43% of traffic offences involved alcohol [17]. In our study, 48% of individuals with BACs ≥ 1.1 g/kg had PEth concentrations above 200 ng/mL, indicating habitual rather than isolated drinking. Extrapolated nationally, approximately 23,000 court evaluations could benefit from combined EtG and PEth testing.

## Limitations/strengths

The duration of alcohol intake, the timing of peak BAC, and the conclusion of the proceedings remain unclear, limiting the precise interpretation of PEth and EtG concentrations.

Furthermore, samples were stored refrigerated in the police station, but transported to Munich without temperature control during a relatively cold period, although in vitro PEth formation in the presence of ethanol at room temperature has been reported in previous studies [18, 19]. Neumann et al. demonstrated that no in vitro formation of PEth in presence of alcohol was observed at storage temperature of 4 °C within a 4 week period. PEth formation in presence of alcohol (1.6 g/l) was also tested at room temperature. After 72 h a mean formation of 54 ng/ml was observed [19]. Since the samples were transported during a relatively cold period PEth formation can be considered to be low.

Despite these limitations, this study uniquely analyzed consecutive blood samples for multiple alcohol biomarkers of real traffic offenses — an approach not routinely performed and permitted only with approval from the Ministry of the Interior of North Rhine-Westphalia.

## Conclusion

The stepwise increase in both EtG and BAC across PEth categories underscores the potential of these biomarkers in aiding forensic differentiation between single-occasion and chronic drinking. These findings highlight the importance of integrating PEth and EtG analyses into routine forensic toxicology to enhance the objectivity of alcohol consumption assessments in court proceedings. When drinking patterns and psychophysical conditions are considered, a meaningful improvement in the accuracy of evaluations – and consequently in traffic safety – can be expected.

The findings suggest that single episodes of excessive alcohol intake account for only a minority of alcohol-related criminal offenses.

## Data Availability

The datasets generated during the current study are available from the corresponding author on reasonable request.
